# Unanticipated stenosis of the distal edge of frozen elephant trunk caused by retrograde perfusion: A case report

**DOI:** 10.1186/s44215-025-00223-4

**Published:** 2025-10-14

**Authors:** Taiki Niki, Jiro Esaki

**Affiliations:** Department of Cardiovascular Surgery, Kobe City Medical Center General Hospital 2-1-1, Minatojimanakamachi, Chuoku, Kobe City, Hyogo, 650-0047 Japan

**Keywords:** Frozenix Partial ET, Acute aortic dissection, Extracorporeal membrane oxygenation

## Abstract

**Background:**

Total arch replacement with frozen elephant trunk (FET) has been reported to be associated with favorable aortic remodeling when used for aortic dissection. However, several complications associated with FET have been reported, including distal stent graft-induced new entry (dSINE), which potentially results in aortic rupture and late mortality. Frozenix Partial ET (Japan Lifeline Inc., Tokyo, Japan) is a unique FET device that has a non-stent zone at the distal 2 cm end to decrease the radial force to lower the incidence of dSINE. Owing to its novelty, there are few preceding literatures regarding its efficacy and complications.

In this report, we present a case of an unanticipated complication arising from the use of Frozenix Partial ET subsequent to the initiation of extracorporeal membrane oxygenation (ECMO).

**Case presentation:**

A 54-year-old male diagnosed with Stanford type A acute aortic dissection underwent emergency total aortic arch replacement, frozen elephant trunk with Frozenix partial ET (Japan Lifeline Inc., Tokyo, Japan), the Bentall procedure, and coronary artery bypass grafting.

The patient developed cardiogenic shock, requiring extracorporeal membrane oxygenation with left common femoral artery and vein cannulation on postoperative day 1. Despite adequate ECMO flow, high doses of catecholamines were required to maintain blood pressure measured at the right radial artery. Transesophageal echocardiography revealed stenosis of the distal part of the FET presumably due to retrograde perfusion from the femoral artery. The addition of left axillary artery cannulation improved systemic circulation, reducing the dose of catecholamine.

**Conclusion:**

Although designed to reduce the risk of dSINE, Frozenix Partial ET may induce unforeseen complications. Particularly, its non-stent distal part can become stenotic under conditions of retrograde perfusion. Surgeons should carefully choose where to cannulate in patients with this device requiring redo surgery or ECMO support.

## Background

The frozen elephant trunk (FET) has been reported to be associated with favorable aortic remodeling when used for acute aortic dissection [1], reducing the need for reintervention on the downstream aorta. FET makes distal aortic anastomosis more proximal in patients with aortic arch aneurysms, resulting in better early operative outcomes. FET also provides a landing zone for possible second-stage endovascular repair or anastomosis sites for possible second-stage open repair in cases of aortic dissection and extensive aortic aneurysm [2].

However, FET is associated with complications, including distal graft-induced new entry (dSINE), spinal cord ischemia, kinking, and endoleak [3].

Frozenix Partial ET (Japan Lifeline Inc., Tokyo, Japan) has a unique feature that the distal 2 cm part of the device is not supported by stent to reduce radial force to mitigate the risk of dSINE and also make future possible second stage anastomosis easier. Despite its potential benefits, there is a paucity of literature evaluating its efficacy and safety.

Herein, we present a case involving an unexpected complication following the device implantation.

## Case presentation

**Fig. 1:**
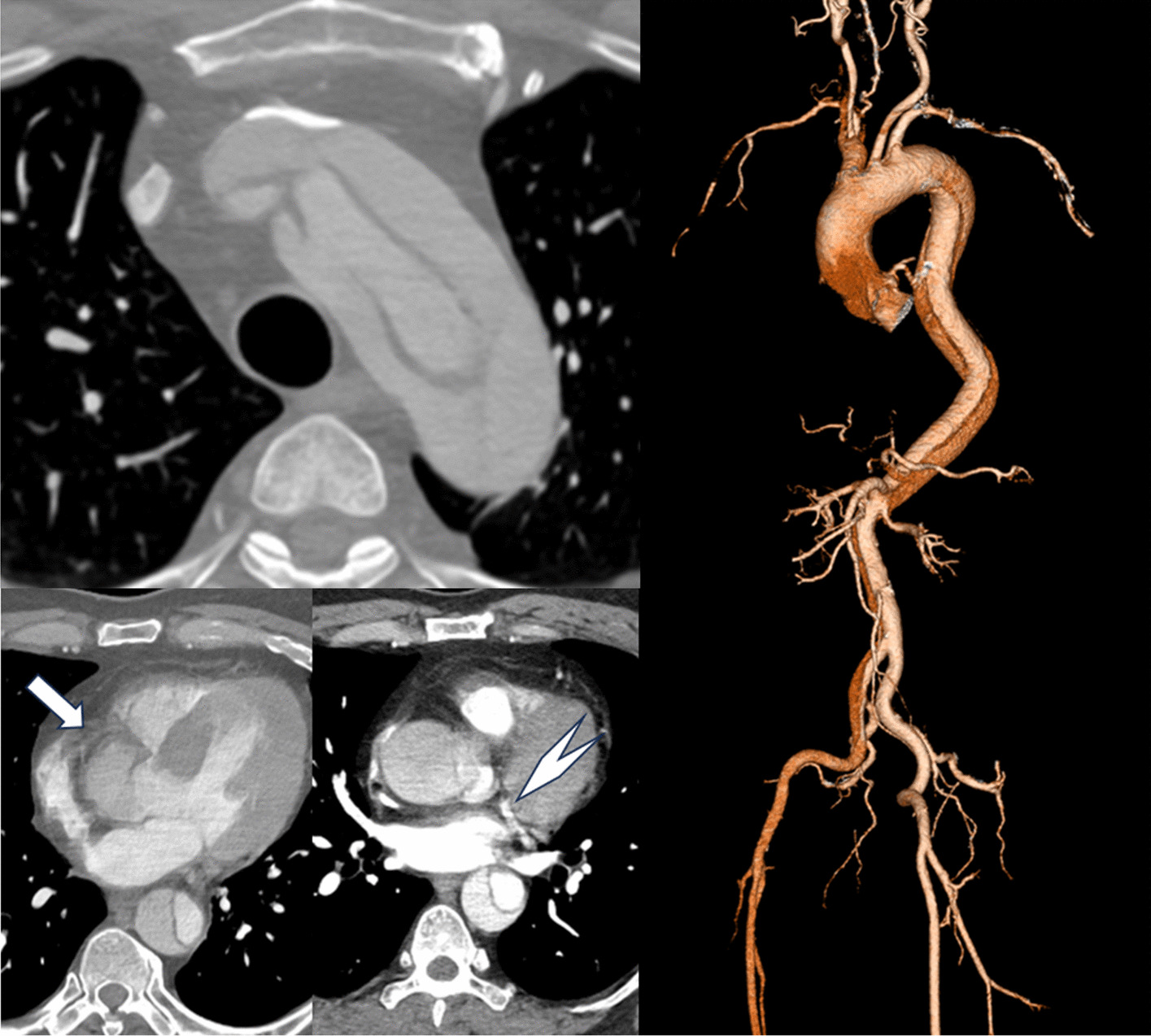
Three-dimensional computed tomography angiography (CTA) on admission. CTA revealed aortic dissection ranging from the aortic root to the iliac arteries. The enhancement of the right coronary artery (arrow) was weaker than that of the left coronary artery (arrowhead)

A 54-year-old male experienced sudden-onset back pain. His vital signs were as follows: blood pressure, 141/99 mmHg; pulse rate, 102/min; and SpO_2_, 97% with 1 L/min oxygen inhalation. Contrast-enhanced computed tomography (CT) revealed acute Stanford type A aortic dissection (ATAAD) extending from the aortic root to the iliac arteries. The primary tear was located in the aortic arch (Fig. 1). The brachiocephalic artery, left common carotid artery, left subclavian artery, celiac artery, and left renal artery originated from both the true and false lumens. Right coronary artery (RCA) enhancement was notably diminished compared to the left coronary artery (Fig. 1). Electrocardiogram did not show any ST change. Preoperative transesophageal echocardiography (TEE) revealed moderate-to-severe aortic regurgitation and pericardial effusion without left ventricular wall motion abnormality.

Emergency surgery was performed via median sternotomy, consisting of total aortic arch replacement, FET, the Bentall procedure, and coronary artery bypass grafting (CABG) to the RCA. The patient underwent total arch replacement with FET since there was a primary tear in the arch and also aortic remodeling potentially achieved by FET was beneficial, considering the patient’s relatively young age. Both the left common femoral artery and left axillary artery were cannulated for cardiopulmonary bypass (CPB). Under circulatory arrest with anterograde cerebral perfusion at moderate hypothermia of 28.0℃, FET with Frozenix partial ET 25*–*60 mm was deployed in the proximal descending aorta. The size of the partial ET was determined according to 90% of the diameter of the straight portion of the descending aorta where distal end of the partial ET was assumed to be located. Total arch replacement was performed with J graft 4 branched 24 mm (Japan Lifeline Inc., Tokyo, Japan). Since the ostium of the right coronary artery was torn probably due to selective cardioplegia administration, Bentall procedure with St. Jude Medical Regent Valve 23 mm (Abbott Inc., St Paul, MN, USA) and Gelweave Valsalva graft 26 mm (Terumo, Ann Arbor, MI, USA) was performed. CABG to the RCA with vein graft was also performed since the proximal part of the RCA was torn.

The patient had difficulties in weaning from CPB because of right ventricular dysfunction, Likely due to insufficient administration of cardioplegia to the RCA prior to CABG. The patient was eventually weaned from CPB with high-dose inotropic support and nitric oxide inhalation. The duration of CPB, aortic cross-clamp, circulatory arrest, and overall surgery were 547 min, 319 min, 64 min, and 736 min, respectively.

On postoperative day (POD) 1, the patient developed cardiac tamponade in addition to right ventricular failure, resulting in a deteriorating circulatory condition. Therefore, the patient underwent re-exploration for bleeding and extracorporeal membrane oxygenation (ECMO) was initiated with left common femoral artery and femoral vein cannulation. No significant blood pressure differences between upper and lower extremities were observed before ECMO introduction. TEE showed that the frozen elephant trunk except for the distal end was dilated with a circular configuration; however, the distal end of the frozen elephant trunk was elliptical rather than circular before ECMO introduction. TEE was not performed after introduction of ECMO.

**Fig. 2:**
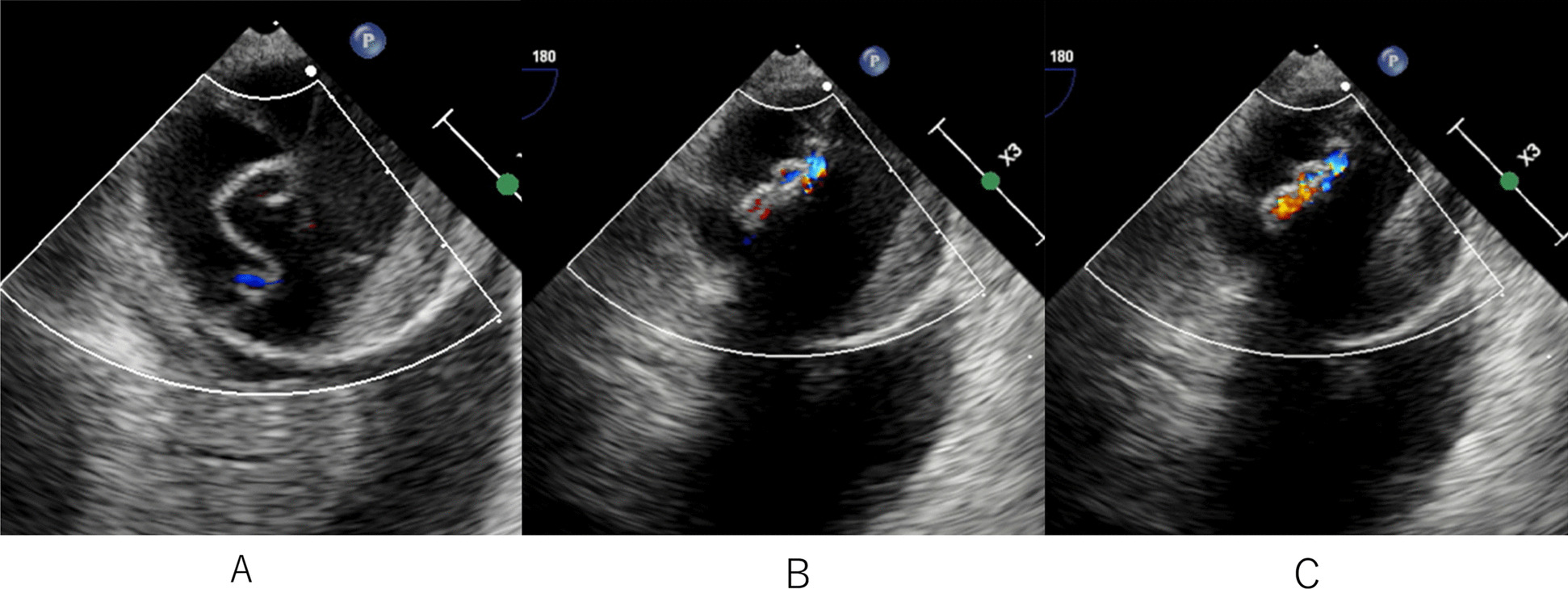
Transesophageal echocardiography (TEE) conducted on postoperative day 2. **A** The true lumen of the aorta was sufficiently dilated and did not compress the partial ET. **B** TEE revealed the stenosis of the distal skirt of Partial ET with 5.0 L/min of ECMO flow. **C** The stenosis was relieved when the ECMO flow was lowered to 2.0 L/min

**Fig. 3:**
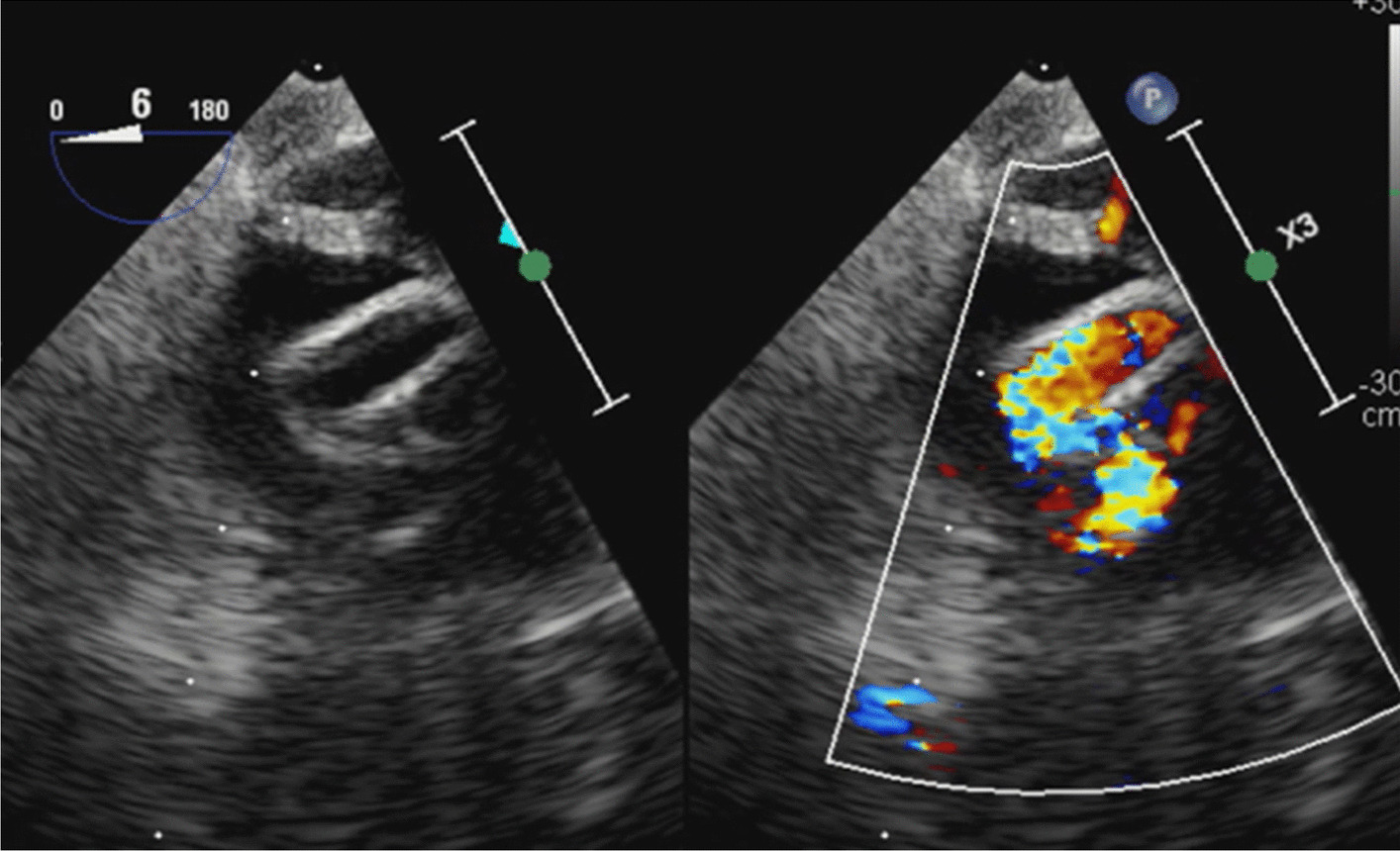
Transesophageal echocardiography (TEE) after an arterial cannula was added to the left axillary artery. After arterial cannula was added to the left axillary artery, the stenosis of the distal skirt of the Partial ET was improved

Although the ECMO flow was stable at 5.0 L/min, high doses of catecholamines were required to Maintain adequate blood pressure monitored via the right radial artery. TEE performed on POD 2 revealed the stenosis of the distal end of the Partial ET, even though the true lumen of the aorta at this level was sufficiently dilated (Fig. 2A, B). Retrograde perfusion from the femoral artery seemed to cause stenosis of the distal end of the FET. Reducing the ECMO flow to 2.0 L/min alleviated the stenosis (Fig. 2C). Therefore, we added left axillary arterial cannulation with the graft (J graft 8 mm, Japan Lifeline Inc., Tokyo, Japan), resulting in a decrease in the dose of catecholamines and a decrease in the level of lactate, along with an improvement of PaO_2_ monitored via the right radial artery and mixed venous oxygen saturation. Furthermore, TEE revealed resolution of the stenosis at the distal end (Fig. 3).

Over the subsequent days, the patient’s circulatory status gradually improved. The patient was weaned from ECMO on POD12. However, shortly after ECMO removal, cardiogenic shock recurred probably due to severe right heart failure. Finally, the patient exhibited pulseless electrical activity and then asystole while preparing for ECMO reintroduction. ECMO was reestablished 20 min after cardiac arrest. The patient ultimately succumbed to refractory cardiogenic shock and multiorgan dysfunction on POD 13.

## Discussion

In acute aortic dissection, FET has been reported to facilitate true lumen expansion and false lumen obliteration, resulting in favorable aortic remodeling and better long-term prognosis with reduced reintervention on the downstream aorta [4], compared with the conventional elephant trunk [1, 5].

FET could also improve abdominal organ malperfusion caused by acute aortic dissection by expanding the true lumen or excluding entry tears in the proximal descending aorta. In addition to acute aortic dissection, the advantages of FET have been documented in other aortic pathologies. FET makes distal aortic anastomosis more proximal in patients with aortic arch aneurysms, which can decrease intraoperative bleeding, shorten the operation time, and improve early operative outcomes [6].

In cases of extensive aortic aneurysm, total arch replacement combined with FET enables the definitive approach as a single-stage procedure [7].

Moreover, if reintervention is needed, FET makes it easier by providing a landing zone for thoracic endovascular aortic repair (TEVAR) or an anastomosis site for open surgery [2].

However, the FET technique is associated with specific complications including spinal cord ischemia, endoleak dSINE, and kinking. Among these, dSINE is particularly concerning, with an incidence reported in approximately 0–27.2% of cases [8–10], typically Manifesting 12–36 months after the procedure [11]. Although asymptomatic in most cases, dSINE is associated with expansion of the false lumen, leading to aneurysm formation and aortic rupture [12], thereby reducing the survival rate [13].

The spring-back force and the radial force of FET are considered contributors to the development of dSINE [14]. Therefore, Frozenix Partial ET has been developed, which has non-stent part at the distal end, reducing the radial force compared with previous FET device. Additionally, its non-stent part at the distal end allows easier anastomosis when two-staged open surgery is needed for the descending aorta.

Despite these design advantages, real-world data on the efficacy and complications of Frozenix Partial ET remains scarce, particularly under conditions involving retrograde perfusion.

In the present case, the stenosis of the distal skirt of the device was identified in TEE during peripheral ECMO support. Either reducing retrograde ECMO flow or augmentation of anterograde flow with left axillary artery cannulation alleviated the stenosis. These findings underscore the unique hemodynamic challenges posed by Frozenix Partial ET under specific perfusion conditions.

To our knowledge, this is the first report which described the compression of elephant trunk (ET) under the retrograde perfusion including conventional elephant trunk. Ohashi et al. described dynamic obstruction of the elephant trunk caused by the vortex flow ascending the exterior of the elephant trunk, resulting in compression of the graft [15]. In their case, TEVAR was performed, effectively resolving the pressure gradient between the ascending aorta and descending aorta.

In a similar manner, our case demonstrated that the stenosis of Frozemix partial ET could arise not from mechanical problems such as kinking of the FET but from hemodynamic forces acting on the device.

If patients who have Frozenix Partial ET need redo cardiac/thoracic aortic surgery or peripheral ECMO support, only retrograde perfusion with femoral artery cannulation is not recommended and antegrade perfusion with aortic or axillary artery cannulation should be considered. Otherwise, TEVAR extension prior to redo surgery might be an option.

Retrospectively, the decision to wean the patient off the ECMO on POD 12 was premature. Although hemodynamics was stable with an ECMO flow of 1.5 L/min, this level of support was critical for the patient given the severe right heart failure.

## Data Availability

Not applicable.

## Abbreviations

FET: Frozen elephant trunk
dSINE: Distal stent graft-induced new entry
CT: Computed tomography
ATAAD: Acute Stanford type A aortic dissection
RCA: Right coronary artery
TEE: Transesophageal echocardiography
CABG: Coronary artery bypass grafting
CPB: Cardiopulmonary bypass
POD: Postoperative day
ECMO: Extracorporeal membrane oxygenation
TEVAR: Thoracic endovascular aortic repair
